# Supplementary material for: Aberrant Resting-State Functional Connectivity in MDD and the Antidepressant Treatment Effect—A 6-Month Follow-Up Study
Source: Brain Sci. 2023 Apr 23;13(5):705. doi: 10.3390/brainsci13050705 (PMC10216051; doi:10.3390/brainsci13050705)
Supplement: Supplementary file 1 [file brainsci-13-00705-s001.zip › brainsci-2317183-supplementary.pdf]

Supplement S1. MRI data acquisition

During the scan session, foam padding and earbuds were used to reduce head movements and attenuate the noise from the scanner. The data acquisition was carried out by trained professionals using the same instructions and the subjects were asked to stay in a quiet and awake state.

A T1-weighted structural image was collected using the spoiled gradient recall sequence with the parameters below: repetition time (TR) = 6.8ms, echo time (TE) = 2.5ms, flip angle = 8°, field of view (FOV) = 256 × 256 mm2, slice thickness = 1mm, slice gap = 0mm, voxel size = 1×1×1mm3, scan time = 6 minutes.

Data of the resting-state functional magnetic resonance (rs-fMRI) were collected using a T2-weighted echo planar imaging sequence (EPI). Before data collection, the subjects were instructed to close their eyes, relax, empty their brains and not think about anything, and keep awake all the time. Parameters were as following: Slice Thickness = 4mm, Slice= 33, Voxel Size = 3.4×3.4×4mm3, Repetition time (TR) = 2000ms, echo time (TE) = 30ms, field of view (FOV) = 220×220mm, flip angle = 90°, number of scans = 180, scan time = 6 minutes.

Table S1. Decreased functional connections in Baseline MDD compared to healthy controls

Brain region1	MNI coordinate (x, y, z)			Brain network	Brain region2	MNI coordinate (x, y, z)			Brain network	Follow-up excluded vs Baseline MDD	Follow-up excluded vs HCs
R frontal eye field	40.7	14.5	48.2	FP	R angular gyrus	54.2	-45.2	36.9	FP	**	ns
R Broca-pars opercularis	40.0	17.6	29.2	FP	R angular gyrus	54.2	-45.2	36.9	FP	*	*
R prefrontal lobe	30.5	54.9	-3.5	FP	R middle temporal gyrus	50.0	-33.8	-0.7	MF	ns	ns
R inferior frontal gyrus, orbital part	53.6	24.8	0.9	MF	R middle temporal gyrus	50.0	-33.8	-0.7	MF	ns	*
R orbitfrontal gyrus	5.1	34.9	-17.4	DMN	R inferior temporal gyrus	40.3	-11.3	-35.8	Mot	ns	**
R primary sensory area	32.4	-39.2	49.6	Mot	R fusiform gyrus	25.2	-44.6	-12.2	VI	ns	*
R prefrontal lobe	44.6	46.2	-4.9	FP	R fusiform gyrus	60.8	-43.3	-17.6	FP	**	ns
R superior temporal gyrus	61.9	-23.8	-2.8	Mot	R visual associative cortex	21.0	-63.7	-9.0	VI	ns	ns
R superior temporal gyrus	61.9	-23.8	-2.8	Mot	R secondary visual cortex	17.9	-83.4	-11.3	VII	ns	*
R superior temporal gyrus	61.9	-23.8	-2.8	Mot	R secondary visual cortex	7.0	-75.7	-2.8	VI	ns	*
R superior temporal gyrus	61.9	-23.8	-2.8	Mot	R secondary visual cortex	31.2	-91.8	-10.8	VII	ns	**
R primary auditory cortex	59.2	-3.4	2.7	Mot	R primary visual cortex	14.6	-68.3	8.3	VI	**	ns
R superior temporal gyrus	61.9	-23.8	-2.8	Mot	R primary visual cortex	14.6	-68.3	8.3	VI	ns	ns
R middle temporal gyrus	50.0	-33.8	-0.7	MF	R dorsal anterior cingulate cortex	7.8	34.7	17.1	SAL	ns	*
R superior temporal gyrus	59.3	-43.8	8.5	MF	R dorsal anterior cingulate cortex	7.8	34.7	17.1	SAL	ns	ns
R visual motor area	14.8	-68.4	34.9	VI	R ventral posterior cingulate cortex	5.1	-38.9	27.0	DMN	ns	*
R superior temporal gyrus	61.9	-23.8	-2.8	Mot	R ventral posterior cingulate cortex	28.4	-53.8	7.1	VI	ns	ns
R supramarginal gyrus	59.0	-29.3	19.5	Mot	R ventral posterior cingulate cortex	7.0	-18.8	29.8	SAL	ns	ns
R superior temporal gyrus	59.3	-43.8	8.5	MF	R ventral posterior cingulate cortex	7.0	-18.8	29.8	SAL	ns	**
R insula	38.3	-12.5	-1.1	Mot	R dorsal posterior cingulate cortex	7.8	-23.1	44.9	Mot	ns	ns
R fusiform gyrus	25.2	-44.6	-12.2	VI	R dorsal posterior cingulate cortex	7.8	-23.1	44.9	Mot	*	*

Brain region1	MNI coordinate (x, y, z)			Brain network	Brain region2	MNI coordinate (x, y, z)			Brain network	Follow-up excluded vs Baseline MDD	Follow-up excluded vs HCs
R superior temporal gyrus	56.5	-8.5	-14.3	MF	R secondary visual cortex	14.6	-46.0	2.8	VI	ns	**
R Broca-pars opercularis	55.4	9.6	22.2	FP	R cerebellum	6.1	-50.7	-12.3	CBL	*	ns
R dorsal posterior cingulate cortex	7.8	-23.1	44.9	Mot	R cerebellum	6.1	-50.7	-12.3	CBL	*	ns
R dorsal posterior cingulate cortex	6.2	-57.4	38.2	DMN	R cerebellum	16.2	-47.2	-52.3	CBL	ns	***
R dorsolateral prefrontal lobe	37.6	35.4	31.1	SAfL	R cerebellum	23.4	-59.3	-52.1	Mot	ns	ns
R frontal eye field	23.9	30.7	36.4	DMN	R cerebellum	7.6	-56.7	-50.8	DMN	ns	*
R pre-supplementary motor area	25.2	12.4	49.4	FP	R cerebellum	7.6	-56.7	-50.8	DMN	*	ns
R pre-supplementary motor area	39.7	3.4	34.0	FP	R caudatum	12.7	2.9	11.5	SC	ns	ns
R prefrontal lobe	28.9	51.1	18.7	FP	R thalamus	5.5	-9.7	5.2	SC	ns	*
R precuneus	7.5	-57.3	61.8	SAL	R thalamus	5.5	-9.7	5.2	SC	ns	ns
R superior temporal gyrus	56.5	-8.5	-14.3	MF	L prefrontal lobe	-6.9	48.3	-5.7	DMN	ns	**
R cerebellum	7.2	-69.0	-20.9	CBL	L prefrontal lobe	-28.8	50.1	21.7	SAL	ns	***
R cerebellum	7.6	-56.7	-50.8	DMN	L prefrontal lobe	-28.8	50.1	21.7	SAL	ns	**
R cerebellum	39.1	-74.9	-29.7	VII	L dorsolateral prefrontal lobe	-10.2	55.7	30.2	MF	ns	*
R cerebellum	7.6	-56.7	-50.8	DMN	L dorsolateral prefrontal lobe	-27.3	34.1	36.4	SAL	ns	***
R cerebellum	39.1	-74.9	-29.7	VII	L frontal eye field	-11.2	34.3	51.5	MF	ns	ns
R fusiform gyrus	60.8	-43.3	-17.6	FP	L frontal eye field	-5.0	17.7	48.1	MF	**	ns
R middle temporal gyrus	50.0	-33.8	-0.7	MF	L inferior frontal gyrus, orbital part	-46.0	28.2	-7.1	MF	**	ns
R superior temporal gyrus	59.3	-43.8	8.5	MF	L inferior frontal gyrus, orbital part	-46.0	28.2	-7.1	MF	**	ns
R dorsolateral prefrontal lobe	37.6	35.4	31.1	SAL	L dorsolateral prefrontal lobe	-43.0	42.0	11.0	FP	ns	ns
R visual associative cortex	21.0	-63.7	-9.0	VI	L Broca-pars opercularis	-53.1	18.4	10.6	MF	ns	ns
R secondary visual cortex	7.0	-75.7	-2.8	VI	L Broca-pars opercularis	-53.1	18.4	10.6	MF	ns	ns

Brain region1	MNI coordinate (x, y, z)			Brain network	Brain region2	MNI coordinate (x, y, z)			Brain network	Follow-up excluded vs Baseline MDD	Follow-up excluded vs HCs
R cerebellum	6.1	-50.7	-12.3	CBL	L Broca-pars opercularis	-53.1	18.4	10.6	MF	**	ns
R cerebellum	7.2	-69.0	-20.9	CBL	L Broca-pars opercularis	-53.1	18.4	10.6	MF	*	ns
R insula	41.4	3.5	7.2	Mot	L primary motor cortex	-41.6	-14.7	44.8	Mot	ns	*
R middle temporal gyrus	50.0	-33.8	-0.7	MF	L primary motor cortex	-41.6	-14.7	44.8	Mot	ns	ns
L primary motor cortex	-41.6	-14.7	44.8	Mot	L pre-supplementary motor area	-57.0	-3.4	6.8	Mot	ns	**
R inferior temporal gyrus	43.4	-26.5	-24.6	VAs	L pre-supplementary motor area	-23.2	10.7	53.6	FP	ns	*
R cerebellum	16.2	-47.2	-52.3	CBL	L pre-supplementary motor area	-23.2	10.7	53.6	FP	ns	ns
R cerebellum	7.6	-56.7	-50.8	DMN	L pre-supplementary motor area	-23.2	10.7	53.6	FP	ns	*
R brainstem	7.5	-34.2	-37.3	CBL	L pre-supplementary motor area	-23.2	10.7	53.6	FP	ns	*
R inferior temporal gyrus	43.4	-26.5	-24.6	VAs	L pre-supplementary motor area	-27.6	-9.1	55.9	Mot	ns	*
R fusiform gyrus	25.2	-44.6	-12.2	VI	L insula	-39.1	1.7	9.5	Mot	ns	**
R cerebellum	6.1	-50.7	-12.3	CBL	L insula	-39.1	1.7	9.5	Mot	ns	**
R pre-supplementary motor area	7.0	-8.1	52.9	Mot	L insula	38.7	8.1	-4.8	SAL	ns	**
R cerebellum	6.1	-50.7	-12.3	CBL	L insula	38.7	8.1	-4.8	SAL	ns	ns
L pre-supplementary motor area	-16.2	-19.2	69.5	Mot	L insula	38.7	8.1	-4.8	SAL	ns	*
L orbitfrontal gyrus	-5.4	29.1	-10.1	DMN	L insula	-37.8	-12.9	-1.4	Mot	*	ns
R fusiform gyrus	25.2	-44.6	-12.2	VI	L primary sensory area	-50.6	-23.8	41.4	Mot	ns	*
R secondary visual cortex	7.8	-88.6	1.9	VI	L primary sensory area	-50.6	-23.8	41.4	Mot	***	ns
R insula	38.3	-12.5	-1.1	Mot	L primary sensory area	-41.2	-15.6	14.5	Mot	ns	ns
R angular gyrus	41.4	-75.3	28.0	DMN	L visual motor area	-9.8	-66.3	55.1	SAL	ns	ns
R cerebellum	6.9	-68.0	-37.3	CBL	L visual motor area	-9.8	-66.3	55.1	SAL	ns	*
R cerebellum	16.2	-47.2	-52.3	CBL	L visual motor area	-9.8	-66.3	55.1	SAL	ns	*

Brain region1	MNI coordinate (x, y, z)			Brain network	Brain region2	MNI coordinate (x, y, z)			Brain network	Follow-up excluded vs Baseline MDD	Follow-up excluded vs HCs
R cerebellum	7.6	-56.7	-50.8	DMN	L visual motor area	-9.8	-66.3	55.1	SAL	***	ns
R cerebellum	41.9	-64.0	-49.2	FP	L visual motor area	-9.8	-66.3	55.1	SAL	ns	ns
R thalamus	5.5	-9.7	5.2	SC	L visual motor area	-9.8	-66.3	55.1	SAL	ns	*
L dorsolateral prefrontal lobe	-27.3	34.1	36.4	SAL	L visual motor area	-9.8	-66.3	55.1	SAL	**	ns
L pre-supplementary motor area	-23.2	10.7	53.6	FP	L visual motor area	-9.8	-66.3	55.1	SAL	ns	ns
R primary visual cortex	14.6	-68.3	8.3	VI	L supramarginal gyrus	-42.2	-31.2	15.9	Mot	ns	*
L primary sensory area	-41.2	-15.6	14.5	Mot	L supramarginal gyrus	-42.2	-31.2	15.9	Mot	ns	**
R fusiform gyrus	25.2	-44.6	-12.2	VI	L supramarginal gyrus	-59.5	-25.9	21.9	Mot	ns	*
L insula	38.7	8.1	-4.8	SAL	L supramarginal gyrus	-59.5	-25.9	21.9	Mot	ns	***
R prefrontal lobe	44.6	46.2	-4.9	FP	L angular gyrus	-53.4	-43.5	38.8	FP	ns	***
R dorsolateral prefrontal lobe	37.6	35.4	31.1	SAL	L angular gyrus	-53.4	-43.5	38.8	FP	ns	ns
R 布 Broca-pars opercularis	40.0	17.6	29.2	FP	L angular gyrus	-53.4	-43.5	38.8	FP	ns	ns
L dorsolateral prefrontal lobe	-43.0	42.0	11.0	FP	L angular gyrus	-53.4	-43.5	38.8	FP	ns	**
R dorsal posterior cingulate cortex	6.2	-57.4	38.2	DMN	L temporal pole	-38.0	6.1	-37.9	MF	ns	*
R primary visual cortex	14.6	-68.3	8.3	VI	L middle temporal gyrus	-49.7	6.4	-15.2	Mot	ns	ns
R cerebellum	6.1	-50.7	-12.3	CBL	L middle temporal gyrus	-49.7	6.4	-15.2	Mot	ns	*
R fusiform gyrus	25.2	-44.6	-12.2	VI	L middle temporal gyrus	-59.0	-30.0	3.5	Mot	ns	**
R visual associative cortex	21.0	-63.7	-9.0	VI	L middle temporal gyrus	-59.0	-30.0	3.5	Mot	**	ns
R secondary visual cortex	7.8	-88.6	1.9	VI	L middle temporal gyrus	-59.0	-30.0	3.5	Mot	ns	ns
R primary visual cortex	14.6	-68.3	8.3	VI	L middle temporal gyrus	-59.0	-30.0	3.5	Mot	ns	ns
R inferior frontal gyrus, orbital part	53.6	24.8	0.9	MF	L inferior temporal gyrus	-57.8	-47.5	5.2	MF	ns	**
R visual associative cortex	21.0	-63.7	-9.0	VI	L inferior temporal gyrus	-51.8	-18.2	-28.8	FP	ns	ns

Brain region1	MNI coordinate (x, y, z)			Brain network	Brain region2	MNI coordinate (x, y, z)			Brain network	Follow-up excluded vs Baseline MDD	Follow-up excluded vs HCs
R primary visual cortex	14.6	-68.3	8.3	VI	L inferior temporal gyrus	-51.8	-18.2	-28.8	FP	ns	**
R dorsolateral prefrontal lobe	37.6	35.4	31.1	SAL	L fusiform gyrus	-60.4	-50.0	-14.0	FP	**	ns
R prefrontal lobe	44.6	46.2	-4.9	FP	L fusiform gyrus	-42.6	-52.1	-17.4	VAs	ns	**
L dorsolateral prefrontal lobe	-43.0	42.0	11.0	FP	L fusiform gyrus	-42.6	-52.1	-17.4	VAs	ns	**
L visual motor area	-9.8	-66.3	55.1	SAL	L visual associative cortex	-41.3	-75.4	22.8	DMN	*	ns
R primary sensory area	32.4	-39.2	49.6	Mot	L visual associative cortex	-17.0	-50.7	0.8	VI	ns	*
R superior temporal gyrus	61.9	-23.8	-2.8	Mot	L visual associative cortex	-17.0	-50.7	0.8	VI	ns	*
R superior temporal gyrus	56.5	-8.5	-14.3	MF	L visual associative cortex	-17.0	-50.7	0.8	VI	ns	***
R dorsal posterior cingulate cortex	7.8	-23.1	44.9	Mot	L visual associative cortex	-17.0	-50.7	0.8	VI	**	ns
R caudatum	13.8	-4.2	20.9	SC	L visual associative cortex	-43.2	-70.4	-13.8	VAs	ns	ns
L dorsolateral prefrontal lobe	-10.2	55.7	30.2	MF	L visual associative cortex	-16.8	-84.9	33.0	VI	ns	*
R superior temporal gyrus	61.9	-23.8	-2.8	Mot	L secondary visual cortex	-36.0	-84.2	-3.9	VAs	ns	ns
R superior temporal gyrus	61.9	-23.8	-2.8	Mot	L secondary visual cortex	-8.9	-70.7	-1.7	VI	ns	ns
L middle temporal gyrus	-59.0	-30.0	3.5	Mot	L secondary visual cortex	-8.9	-70.7	-1.7	VI	*	ns
L frontal eye field	-11.2	34.3	51.5	MF	L secondary visual cortex	-14.7	-84.0	-13.1	VII	ns	**
R superior temporal gyrus	61.9	-23.8	-2.8	Mot	L primary visual cortex	-22.1	-66.7	7.5	VI	ns	ns
L frontal eye field	-11.2	34.3	51.5	MF	L primary visual cortex	-22.1	-66.7	7.5	VI	ns	**
R insula	38.3	-12.5	-1.1	Mot	L pre-supplementary motor area	-7.8	-22.4	46.1	Mot	ns	*
L insula	-37.8	-12.9	-1.4	Mot	L pre-supplementary motor area	-7.8	-22.4	46.1	Mot	ns	ns
L angular gyrus	-53.4	-43.5	38.8	FP	L dorsal anterior cingulate cortex	-6.0	34.1	26.3	MF	ns	**
R visual motor area	14.8	-68.4	34.9	VI	L ventral posterior cingulate cortex	-5.0	-36.0	32.0	DMN	*	ns
R visual motor area	14.8	-68.4	34.9	VI	L ventral posterior cingulate cortex	-7.4	-18.2	30.0	SAL	**	ns

Brain region1	MNI coordinate (x, y, z)			Brain network	Brain region2	MNI coordinate (x, y, z)			Brain network	Follow-up excluded vs Baseline MDD	Follow-up excluded vs HCs
R superior temporal gyrus	59.3	-43.8	8.5	MF	L ventral posterior cingulate cortex	-7.4	-18.2	30.0	SAL	ns	*
R cerebellum	16.2	-47.2	-52.3	CBL	L ventral posterior cingulate cortex	-6.5	-53.9	37.4	DMN	ns	***
R cerebellum	7.6	-56.7	-50.8	DMN	L ventral posterior cingulate cortex	-6.5	-53.9	37.4	DMN	ns	**
R dorsal posterior cingulate cortex	6.2	-57.4	38.2	DMN	L posterior limb	-7.5	-42.1	13.3	DMN	ns	**
L visual motor area	-9.5	-71.0	31.9	VI	L posterior limb	-7.5	-42.1	13.3	DMN	ns	**
L visual motor area	-9.8	-66.3	55.1	SAL	L posterior limb	-7.5	-42.1	13.3	DMN	ns	*
L ventral posterior cingulate cortex	-6.5	-53.9	37.4	DMN	L posterior limb	-7.5	-42.1	13.3	DMN	ns	**
R pre-supplementary motor area	7.0	-8.1	52.9	Mot	L amygdala	-26.8	2.4	-18.7	Mot	**	ns
L pre-supplementary motor area	-16.2	-19.2	69.5	Mot	L amygdala	-26.8	2.4	-18.7	Mot	ns	ns
L dorsolateral prefrontal lobe	-27.3	34.1	36.4	SAL	L hippocampus	-21.5	-36.9	5.8	SC	*	ns
L visual motor area	-9.8	-66.3	55.1	SAL	L hippocampus	-21.5	-36.9	5.8	SC	*	ns
L visual motor area	-9.8	-66.3	55.1	SAL	L hippocampus	-32.1	-40.2	-4.0	SC	ns	ns
R secondary visual cortex	7.7	-75.0	25.0	VI	L hippocampus	-35.7	-24.8	-14.9	SC	ns	ns
R dorsal posterior cingulate cortex	6.2	-57.4	38.2	DMN	L hippocampus	-35.7	-24.8	-14.9	SC	ns	ns
L frontal eye field	-11.2	34.3	51.5	MF	L hippocampus	-35.7	-24.8	-14.9	SC	ns	ns
L ventral posterior cingulate cortex	-6.5	-53.9	37.4	DMN	L hippocampus	-35.7	-24.8	-14.9	SC	*	ns
R pre-supplementary motor area	25.2	12.4	49.4	FP	L cerebellum	-8.7	-50.6	-39.6	CBL	ns	ns
L dorsal posterior cingulate cortex	-9.0	-42.6	50.1	SAL	L cerebellum	-8.7	-50.6	-39.6	CBL	ns	*
R frontal eye field	23.9	30.7	36.4	DMN	L cerebellum	-8.7	-55.2	-52.1	DMN	ns	ns
R pre-supplementary motor area	25.2	12.4	49.4	FP	L cerebellum	-8.7	-55.2	-52.1	DMN	*	ns
R dorsal posterior cingulate cortex	6.2	-57.4	38.2	DMN	L cerebellum	-8.7	-55.2	-52.1	DMN	ns	*
L pre-supplementary motor area	-23.2	10.7	53.6	FP	L cerebellum	-8.7	-55.2	-52.1	DMN	ns	*

Brain region1	MNI coordinate (x, y, z)			Brain network	Brain region2	MNI coordinate (x, y, z)			Brain network	Follow-up excluded vs Baseline MDD	Follow-up excluded vs HCs
L visual motor area	-9.8	-66.3	55.1	SAL	L cerebellum	-8.7	-55.2	-52.1	DMN	*	ns
L ventral posterior cingulate cortex	-6.5	-53.9	37.4	DMN	L cerebellum	-8.7	-55.2	-52.1	DMN	ns	**
R dorsolateral prefrontal lobe	37.6	35.4	31.1	SAL	L cerebellum	-21.2	-70.1	-48.9	VAs	ns	ns
R dorsolateral prefrontal lobe	48.3	35.7	15.2	FP	L cerebellum	-21.2	-70.1	-48.9	VAs	ns	ns
R Broca-pars opercularis	55.4	9.6	22.2	FP	L cerebellum	-21.2	-70.1	-48.9	VAs	ns	*
R pre-supplementary motor area	32.1	-5.4	52.1	SAL	L cerebellum	-21.2	-70.1	-48.9	VAs	ns	ns
R fusiform gyrus	55.2	-56.3	-4.8	VAs	L cerebellum	-21.2	-70.1	-48.9	VAs	ns	*
R dorsolateral prefrontal lobe	8.4	53.3	23.9	MF	L cerebellum	-40.3	-74.2	-29.2	VII	ns	**
R frontal eye field	14.3	36.9	48.9	MF	L cerebellum	-40.3	-74.2	-29.2	VII	ns	**
R frontal eye field	23.9	30.7	36.4	DMN	L cerebellum	-40.3	-74.2	-29.2	VII	ns	***
R frontal eye field	40.7	14.5	48.2	FP	L cerebellum	-40.3	-74.2	-29.2	VII	ns	**
R Broca-pars opercularis	40.0	17.6	29.2	FP	L cerebellum	-40.3	-74.2	-29.2	VII	ns	***
R pre-supplementary motor area	25.2	12.4	49.4	FP	L cerebellum	-40.3	-74.2	-29.2	VII	ns	*
R angular gyrus	47.8	-61.9	34.7	FP	L cerebellum	-40.3	-74.2	-29.2	VII	ns	*
R angular gyrus	41.4	-75.3	28.0	DMN	L cerebellum	-40.3	-74.2	-29.2	VII	*	ns
L prefrontal lobe	-6.0	48.1	11.7	MF	L cerebellum	-40.3	-74.2	-29.2	VII	ns	**
L prefrontal lobe	-11.7	65.1	4.2	DMN	L cerebellum	-40.3	-74.2	-29.2	VII	ns	ns
L dorsolateral prefrontal lobe	-10.2	55.7	30.2	MF	L cerebellum	-40.3	-74.2	-29.2	VII	ns	*
L frontal eye field	-11.2	34.3	51.5	MF	L cerebellum	-40.3	-74.2	-29.2	VII	ns	*
L angular gyrus	-42.1	-65.6	41.7	FP	L cerebellum	-40.3	-74.2	-29.2	VII	*	ns
R dorsolateral prefrontal lobe	48.3	35.7	15.2	FP	L cerebellum	-6.5	-50.1	-11.4	SAL	***	ns
R frontal eye field	23.9	30.7	36.4	DMN	L cerebellum	-27.8	-36.0	-30.9	SAL	ns	*

Brain region1	MNI coordinate (x, y, z)			Brain network	Brain region2	MNI coordinate (x, y, z)			Brain network	Follow-up excluded vs Baseline MDD	Follow-up excluded vs HCs
R dorsal posterior cingulate cortex	6.2	-57.4	38.2	DMN	L cerebellum	-27.8	-36.0	-30.9	SAL	ns	**
R dorsolateral prefrontal lobe	37.6	35.4	31.1	SAL	L cerebellum	-42.6	-63.7	-46.3	FP	ns	*
R frontal eye field	40.7	14.5	48.2	FP	L cerebellum	-42.6	-63.7	-46.3	FP	*	ns
R pre-supplementary motor area	32.1	-5.4	52.1	SAL	L cerebellum	-42.6	-63.7	-46.3	FP	ns	*
R frontal eye field	40.7	14.5	48.2	FP	L cerebellum	-10.3	-81.2	-32.3	FP	ns	ns
R Broca-pars opercularis	40.0	17.6	29.2	FP	L cerebellum	-10.3	-81.2	-32.3	FP	ns	*
R pre-supplementary motor area	6.1	14.0	48.7	SAL	L cerebellum	-10.3	-81.2	-32.3	FP	ns	*
R pre-supplementary motor area	25.2	12.4	49.4	FP	L cerebellum	-10.3	-81.2	-32.3	FP	ns	ns
R pre-supplementary motor area	39.7	3.4	34.0	FP	L cerebellum	-10.3	-81.2	-32.3	FP	ns	ns
R angular gyrus	47.8	-61.9	34.7	FP	L cerebellum	-10.3	-81.2	-32.3	FP	ns	ns
L prefrontal lobe	-28.8	50.1	21.7	SAL	L cerebellum	-8.0	-68.4	-19.9	CBL	ns	*
L dorsolateral prefrontal lobe	-43.0	42.0	11.0	FP	L cerebellum	-8.0	-68.4	-19.9	CBL	ns	ns
L Broca-pars opercularis	-53.1	18.4	10.6	MF	L cerebellum	-8.0	-68.4	-19.9	CBL	*	ns
R prefrontal lobe	44.6	46.2	-4.9	FP	L cerebellum	-26.3	-69.5	-30.6	CBL	ns	ns
R pre-supplementary motor area	6.1	14.0	48.7	SAL	L cerebellum	-26.3	-69.5	-30.6	CBL	ns	***
R pre-supplementary motor area	39.7	3.4	34.0	FP	L cerebellum	-26.3	-69.5	-30.6	CBL	ns	**
R pre-supplementary motor area	32.1	-5.4	52.1	SAL	L cerebellum	-26.3	-69.5	-30.6	CBL	ns	*
L Broca-pars opercularis	-53.1	18.4	10.6	MF	L cerebellum	-26.3	-69.5	-30.6	CBL	ns	ns
L pre-supplementary motor area	-23.2	10.7	53.6	FP	L cerebellum	-24.3	-37.8	-44.3	CBL	**	ns
R fusiform gyrus	46.5	-59.9	-14.8	VAs	L caudatum	-14.6	-3.5	21.1	SC	ns	ns
L visual associative cortex	-43.2	-70.4	-13.8	VAs	L caudatum	-14.6	-3.5	21.1	SC	ns	ns
L cerebellum	-40.3	-74.2	-29.2	VII	L caudatum	-14.6	-3.5	21.1	SC	ns	*

Brain region1	MNI coordinate (x, y, z)			Brain network	Brain region2	MNI coordinate (x, y, z)			Brain network	Follow-up excluded vs Baseline MDD	Follow-up excluded vs HCs
R fusiform gyrus	46.5	-59.9	-14.8	VAs	L putamen	-24.8	5.6	-0.1	SC	ns	*
R fusiform gyrus	55.2	-56.3	-4.8	VAs	L putamen	-24.8	5.6	-0.1	SC	ns	*
R visual associative cortex	21.0	-63.7	-9.0	VI	L putamen	-24.8	5.6	-0.1	SC	*	ns
R visual associative cortex	45.1	74.3	2.6	VAs	L putamen	-24.8	5.6	-0.1	SC	ns	**
R secondary visual cortex	7.8	-88.6	1.9	VI	L putamen	-24.8	5.6	-0.1	SC	ns	*
R precuneus	7.5	-57.3	61.8	SAL	L thalamus	-4.9	-10.4	5.8	SC	ns	*
L visual motor area	-9.8	-66.3	55.1	SAL	L thalamus	-4.9	-10.4	5.8	SC	ns	**
R pre-supplementary motor area	7.0	-8.1	52.9	Mot	L thalamus	-11.6	-25.6	14.8	SC	*	ns
R precuneus	7.5	-57.3	61.8	SAL	L thalamus	-11.6	-25.6	14.8	SC	ns	ns
R ventral anterior cingulate cortex	5.3	-1.0	35.6	Mot	L thalamus	-11.6	-25.6	14.8	SC	*	*
L pre-supplementary motor area	-6.5	-4.3	47.6	Mot	L thalamus	-11.6	-25.6	14.8	SC	ns	ns
L pre-supplementary motor area	-27.6	-9.1	55.9	Mot	L thalamus	-11.6	-25.6	14.8	SC	*	ns
L visual motor area	-9.8	-66.3	55.1	SAL	L thalamus	-11.6	-25.6	14.8	SC	ns	ns
L pre-supplementary motor area	-7.8	-22.4	46.1	Mot	L thalamus	-11.6	-25.6	14.8	SC	ns	*
L dorsal posterior cingulate cortex	-9.0	-42.6	50.1	SAL	L thalamus	-11.6	-25.6	14.8	SC	*	ns

L, left. R, right. MF, medial fronntal network. FP, frontal-parietal. DMN, default mode network. Mot, Motor network. VI, visual networkI. VII, visual networkII. VAs, visual association network. SAL, Salience network. Subcortical network, SC. CBL, cerebellum network. ns, no significant difference. * $P < 0.05$, ** $P < 0.01$, *** $P < 0.001$. HCs, healthy controls.

Table S2. Increased functional connections in baseline MDD compared to healthy controls.

Brain region1	MNI coordinate (x, y, z)			Brain network	Brain region2	MNI coordinate (x, y, z)			Brain network	Follow-up excluded vs baseline MDD	Follow-up excluded vs HCs
R dorsolateral prefrontal lobe	37.6	35.4	31.1	SAL	R frontal eye field	23.9	30.7	36.4	DMN	ns	**
R orbitfrontal gyrus	5.1	34.9	-17.4	DMN	R frontal eye field	40.7	14.5	48.2	FP	**	ns
R prefrontal lobe	8.2	45.9	-1.7	DMN	R frontal eye field	40.7	14.5	48.2	FP	**	ns
R prefrontal lobe	8.2	45.9	-1.7	DMN	R Broca-pars opercularis	40	17.6	29.2	FP	ns	*
R frontal eye field	23.9	30.7	36.4	DMN	R pre-supplementary motor area	6.1	14	48.7	SAL	ns	**
R dorsolateral prefrontal lobe	8.4	53.3	23.9	MF	R pre-supplementary motor area	32.1	-5.4	52.1	SAL	ns	**
R pre-supplementary motor area	6	-22.3	65.6	Mot	R primary sensory area	32.4	-39.2	49.6	Mot	**	ns
R primary sensory area	32.4	-39.2	49.6	Mot	R precuneus	7.5	-57.3	61.8	SAL	**	ns
R precuneus	7.5	-57.3	61.8	SAL	R superior temporal gyrus	61.9	-23.8	-2.8	Mot	ns	**
R pre-supplementary motor area	13.7	6.3	65.4	SAL	R visual associative cortex	21	-63.7	-9	VI	*	ns
R pre-supplementary motor area	39.7	3.4	34	FP	R visual associative cortex	21	-63.7	-9	VI	ns	*
R prefrontal lobe	44.6	46.2	-4.9	FP	R secondary visual cortex	7.7	-75	25	VI	ns	*
R Broca-pars opercularis	40	17.6	29.2	FP	R secondary visual cortex	7.7	-75	25	VI	ns	ns
R Broca-pars opercularis	40	17.6	29.2	FP	R primary visual cortex	14.6	-68.3	8.3	VI	ns	ns
R visual motor area	14.8	-68.4	34.9	VI	R primary visual cortex	14.6	-68.3	8.3	VI	ns	ns
R prefrontal lobe	44.6	46.2	-4.9	FP	R ventral posterior cingulate cortex	12.3	-57.2	18.1	DMN	ns	***
R precuneus	7.5	-57.3	61.8	SAL	R dorsal posterior cingulate cortex	7.8	-23.1	44.9	Mot	***	ns
R cerebellum	6.1	-50.7	-12.3	CBL	R cerebellum	39.1	-74.9	-29.7	VII	ns	ns
R secondary visual cortex	7	-75.7	-2.8	VI	R cerebellum	11.7	84.1	-34.7	FP	ns	ns
R secondary visual cortex	17.9	-83.4	-11.3	VII	R cerebellum	7.6	-56.7	-50.8	DMN	ns	ns

Brain region1	MNI coordinate (x, y, z)			Brain network	Brain region2	MNI coordinate (x, y, z)			Brain network	Follow-up excluded vs baseline MDD	Follow-up excluded vs HCs
R superior temporal gyrus	61.9	-23.8	-2.8	Mot	R thalamus	5.5	-9.7	5.2	SC	ns	*
R frontal eye field	40.7	14.5	48.2	FP	L prefrontal lobe	-6.9	48.3	-5.7	DMN	**	ns
R dorsolateral prefrontal lobe	37.6	35.4	31.1	SAL	L prefrontal lobe	-6	48.1	11.7	MF	**	ns
R dorsolateral prefrontal lobe	48.3	35.7	15.2	FP	L prefrontal lobe	-6	48.1	11.7	MF	*	ns
R pre-supplementary motor area	32.1	-5.4	52.1	SAL	L prefrontal lobe	-6	48.1	11.7	MF	ns	*
R visual motor area	31.6	-60.9	49.2	Vas	L prefrontal lobe	-6	48.1	11.7	MF	ns	*
R dorsal posterior cingulate cortex	7.8	-23.1	44.9	Mot	L prefrontal lobe	-28.8	50.1	21.7	SAL	*	ns
L orbitfrontal gyrus	-5.4	29.1	-10.1	DMN	L prefrontal lobe	-28.8	50.1	21.7	SAL	**	ns
R pre-supplementary motor area	32.1	-5.4	52.1	SAL	L dorsolateral prefrontal lobe	-10.2	55.7	30.2	MF	ns	***
R dorsolateral prefrontal lobe	37.6	35.4	31.1	SAL	L dorsolateral prefrontal lobe	-27.3	34.1	36.4	SAL	ns	*
L orbitfrontal gyrus	-5.4	29.1	-10.1	DMN	L dorsolateral prefrontal lobe	-27.3	34.1	36.4	SAL	ns	ns
R dorsolateral prefrontal lobe	37.6	35.4	31.1	SAL	L frontal eye field	-11.2	34.3	51.5	MF	ns	ns
R secondary visual cortex	7.7	-75	25	VI	L dorsolateral prefrontal lobe	-43	42	11	FP	ns	ns
R cerebellum	16.2	-47.2	-52.3	CBL	L pre-supplementary motor area	-57	-3.4	6.8	Mot	ns	*
L inferior frontal gyrus, orbital part	-46	28.2	-7.1	MF	L pre-supplementary motor area	-23.2	10.7	53.6	FP	**	ns
L orbitfrontal gyrus	-5.4	29.1	-10.1	DMN	L pre-supplementary motor area	-27.6	-9.1	55.9	Mot	***	ns
R cerebellum	39.1	-74.9	-29.7	VII	L insula	-39.1	1.7	9.5	Mot	ns	**
R cerebellum	7.6	-56.7	-50.8	DMN	L insula	-37.8	-12.9	-1.4	Mot	ns	ns
L Broca-pars opercularis	-53.1	18.4	10.6	MF	L visual motor area	-9.5	-71	31.9	VI	ns	ns
R pre-supplementary motor area	7	-8.1	52.9	Mot	L precuneus	-9.8	-66.3	55.1	SAL	ns	*
R pre-supplementary motor area	39.7	3.4	34	FP	L precuneus	-9.8	-66.3	55.1	SAL	ns	*

Brain region1	MNI coordinate (x, y, z)			Brain network	Brain region2	MNI coordinate (x, y, z)			Brain network	Follow-up excluded vs baseline MDD	Follow-up excluded vs HCs
R primary sensory area	32.4	-39.2	49.6	Mot	L precuneus	-9.8	-66.3	55.1	SAL	**	*
R primary auditory cortex	59.2	-3.4	2.7	Mot	L precuneus	-9.8	-66.3	55.1	SAL	ns	**
L pre-supplementary motor area	-58.1	-5.6	27.2	Mot	L precuneus	-9.8	-66.3	55.1	SAL	*	ns
L pre-supplementary motor area	-57	-3.4	6.8	Mot	L precuneus	-9.8	-66.3	55.1	SAL	ns	*
L visual motor area	-25.4	-54.8	64.1	Vas	L precuneus	-9.8	-66.3	55.1	SAL	*	ns
R cerebellum	41.9	-64	-49.2	FP	L supramarginal gyrus	-42.2	-31.2	15.9	Mot	ns	*
R cerebellum	39.1	-74.9	-29.7	VII	L supramarginal gyrus	-59.5	-25.9	21.9	Mot	*	ns
R orbitfrontal gyrus	5.1	34.9	-17.4	DMN	L angular gyrus	-42.1	-65.6	41.7	FP	*	ns
R orbitfrontal gyrus	15.6	34.1	-22.6	FP	L angular gyrus	-42.1	-65.6	41.7	FP	ns	*
R pre-supplementary motor area	7	-8.1	52.9	Mot	L angular gyrus	-42.1	-65.6	41.7	FP	ns	*
L orbitfrontal gyrus	-18.2	19.1	-21	SAL	L angular gyrus	-42.1	-65.6	41.7	FP	*	ns
L inferior frontal gyrus, orbital part	-28.4	36	-15.6	SAL	L angular gyrus	-42.1	-65.6	41.7	FP	*	ns
R cerebellum	41.9	-64	-49.2	FP	L middle temporal gyrus	-59	-30	3.5	Mot	ns	ns
R thalamus	5.5	-9.7	5.2	SC	L middle temporal gyrus	-59	-30	3.5	Mot	ns	***
L precuneus	-9.8	-66.3	55.1	SAL	L middle temporal gyrus	-59	-30	3.5	Mot	ns	**
R orbitfrontal gyrus	5.1	34.9	-17.4	DMN	L visual associative cortex	-41.3	-75.4	22.8	DMN	**	ns
R Broca-pars opercularis	40	17.6	29.2	FP	L visual associative cortex	-41.3	-75.4	22.8	DMN	ns	*
L orbitfrontal gyrus	-5.4	29.1	-10.1	DMN	L visual associative cortex	-41.3	-75.4	22.8	DMN	*	ns
R orbitfrontal gyrus	5.1	34.9	-17.4	DMN	L visual associative cortex	-16.8	-84.9	33	VI	ns	ns
R Broca-pars opercularis	40	17.6	29.2	FP	L visual associative cortex	-16.8	-84.9	33	VI	ns	ns
L orbitfrontal gyrus	-5.4	29.1	-10.1	DMN	L visual associative cortex	-16.8	-84.9	33	VI	ns	ns

Brain region1	MNI coordinate (x, y, z)			Brain network	Brain region2	MNI coordinate (x, y, z)			Brain network	Follow-up excluded vs baseline MDD	Follow-up excluded vs HCs
L orbitfrontal gyrus	-8.2	39.7	-21.4	MF	L visual associative cortex	-16.8	-84.9	33	VI	ns	ns
L precuneus	-9.8	-66.3	55.1	SAL	L pre-supplementary motor area	-7.8	-22.4	46.1	Mot	*	ns
L dorsolateral prefrontal lobe	-10.2	55.7	30.2	MF	L dorsal anterior cingulate cortex	-6	34.1	26.3	MF	*	*
L frontal eye field	-11.2	34.3	51.5	MF	L dorsal anterior cingulate cortex	-6	34.1	26.3	MF	*	ns
R prefrontal lobe	44.6	46.2	-4.9	FP	L ventral posterior cingulate cortex	-8.6	-58.8	17.6	DMN	ns	**
R prefrontal lobe	44.6	46.2	-4.9	FP	L ventral posterior cingulate cortex	-5	-36	32	DMN	ns	*
R dorsolateral prefrontal lobe	48.3	35.7	15.2	FP	L ventral posterior cingulate cortex	-6.5	-53.9	37.4	DMN	ns	ns
R Broca-pars opercularis	40	17.6	29.2	FP	L ventral posterior cingulate cortex	-6.5	-53.9	37.4	DMN	ns	*
R pre-supplementary motor area	6.1	14	48.7	SAL	L ventral posterior cingulate cortex	-6.5	-53.9	37.4	DMN	ns	**
L middle temporal gyrus	-59	-30	3.5	Mot	L posterior limb	-7.5	-42.1	13.3	DMN	ns	ns
L fusiform gyrus	-42.6	-52.1	-17.4	Vas	L hippocampus	-32.1	-40.2	-4	SC	ns	***
R secondary visual cortex	17.9	-83.4	-11.3	VII	L cerebellum	-8.7	-50.6	-39.6	CBL	ns	**
L fusiform gyrus	-42.6	-52.1	-17.4	Vas	L cerebellum	-8.7	-50.6	-39.6	CBL	ns	***
L visual associative cortex	-43.2	-70.4	-13.8	Vas	L cerebellum	-8.7	-50.6	-39.6	CBL	ns	**
L secondary visual cortex	-14.7	-84	-13.1	VII	L cerebellum	-8.7	-50.6	-39.6	CBL	ns	ns
L visual associative cortex	-43.2	-70.4	-13.8	Vas	L cerebellum	-37	-52.9	-31.1	CBL	ns	**
L pre-supplementary motor area	-57	-3.4	6.8	Mot	L cerebellum	-8.7	-55.2	-52.1	DMN	ns	**
L insula	-37.8	-12.9	-1.4	Mot	L cerebellum	-8.7	-55.2	-52.1	DMN	ns	ns
L supramarginal gyrus	-42.2	-31.2	15.9	Mot	L cerebellum	-8.7	-55.2	-52.1	DMN	ns	*
R insula	38.3	-12.5	-1.1	Mot	L cerebellum	-40.3	-74.2	-29.2	VII	ns	**
R supramarginal gyrus	59	-29.3	19.5	Mot	L cerebellum	-40.3	-74.2	-29.2	VII	*	ns

Brain region1	MNI coordinate (x, y, z)			Brain network	Brain region2	MNI coordinate (x, y, z)			Brain network	Follow-up excluded vs baseline MDD	Follow-up excluded vs HCs
R primary auditory cortex	39.9	-25.6	14.4	Mot	L cerebellum	-40.3	-74.2	-29.2	VII	ns	***
R fusiform gyrus	46.5	-59.9	-14.8	Vas	L cerebellum	-40.3	-74.2	-29.2	VII	ns	**
R fusiform gyrus	36.5	-69.1	-17.5	Vas	L cerebellum	-40.3	-74.2	-29.2	VII	ns	ns
R visual associative cortex	21	-63.7	-9	VI	L cerebellum	-40.3	-74.2	-29.2	VII	ns	ns
R cerebellum	6.1	-50.7	-12.3	CBL	L cerebellum	-40.3	-74.2	-29.2	VII	ns	ns
R cerebellum	21.1	-54.8	-23.8	SAL	L cerebellum	-40.3	-74.2	-29.2	VII	ns	*
L pre-supplementary motor area	-57	-3.4	6.8	Mot	L cerebellum	-40.3	-74.2	-29.2	VII	ns	ns
L insula	-39.1	1.7	9.5	Mot	L cerebellum	-40.3	-74.2	-29.2	VII	ns	**
L insula	-37.8	-12.9	-1.4	Mot	L cerebellum	-40.3	-74.2	-29.2	VII	ns	*
L primary sensory area	-41.2	-15.6	14.5	Mot	L cerebellum	-40.3	-74.2	-29.2	VII	ns	*
L supramarginal gyrus	-42.2	-31.2	15.9	Mot	L cerebellum	-40.3	-74.2	-29.2	VII	ns	**
L supramarginal gyrus	-59.5	-25.9	21.9	Mot	L cerebellum	-40.3	-74.2	-29.2	VII	ns	**
L middle temporal gyrus	-59	-30	3.5	Mot	L cerebellum	-40.3	-74.2	-29.2	VII	ns	*
L fusiform gyrus	-42.6	-52.1	-17.4	Vas	L cerebellum	-40.3	-74.2	-29.2	VII	ns	**
L visual associative cortex	-25.9	-63.1	-12.3	VI	L cerebellum	-40.3	-74.2	-29.2	VII	ns	*
R primary auditory cortex	39.9	-25.6	14.4	Mot	L cerebellum	-30.2	-80.2	-40.4	FP	ns	ns
R cerebellum	6.1	-50.7	-12.3	CBL	L cerebellum	-42.6	-63.7	-46.3	FP	***	ns
L primary sensory area	-41.2	-15.6	14.5	Mot	L cerebellum	-42.6	-63.7	-46.3	FP	ns	*
L middle temporal gyrus	-59	-30	3.5	Mot	L cerebellum	-42.6	-63.7	-46.3	FP	ns	ns
L visual associative cortex	-25.9	-63.1	-12.3	VI	L cerebellum	-42.6	-63.7	-46.3	FP	ns	ns
L secondary visual cortex	-36	-84.2	-3.9	Vas	L cerebellum	-42.6	-63.7	-46.3	FP	ns	ns

Brain region1	MNI coordinate (x, y, z)			Brain network	Brain region2	MNI coordinate (x, y, z)			Brain network	Follow-up excluded vs baseline MDD	Follow-up excluded vs HCs
L cerebellum	-6.5	-50.1	-11.4	SAL	L cerebellum	-42.6	-63.7	-46.3	FP	*	ns
R fusiform gyrus	36.5	-69.1	-17.5	Vas	L cerebellum	-10.3	-81.2	-32.3	FP	ns	ns
R visual associative cortex	21	-63.7	-9	VI	L cerebellum	-10.3	-81.2	-32.3	FP	ns	ns
R secondary visual cortex	17.9	-83.4	-11.3	VII	L cerebellum	-10.3	-81.2	-32.3	FP	*	ns
R secondary visual cortex	7	-75.7	-2.8	VI	L cerebellum	-10.3	-81.2	-32.3	FP	ns	ns
R primary visual cortex	14.6	-68.3	8.3	VI	L cerebellum	-10.3	-81.2	-32.3	FP	ns	ns
R cerebellum	6.1	-50.7	-12.3	CBL	L cerebellum	-10.3	-81.2	-32.3	FP	ns	*
L visual associative cortex	-25.9	-63.1	-12.3	VI	L cerebellum	-10.3	-81.2	-32.3	FP	ns	**
L secondary visual cortex	-8.9	-70.7	-1.7	VI	L cerebellum	-10.3	-81.2	-32.3	FP	ns	*
L secondary visual cortex	-14.7	-84	-13.1	VII	L cerebellum	-10.3	-81.2	-32.3	FP	ns	ns
R secondary visual cortex	17.9	-83.4	-11.3	VII	L cerebellum	-8	-68.4	-19.9	CBL	*	ns
R secondary visual cortex	17.9	-83.4	-11.3	VII	L cerebellum	-26.3	-69.5	-30.6	CBL	*	ns
R secondary visual cortex	7	-75.7	-2.8	VI	L cerebellum	-26.3	-69.5	-30.6	CBL	ns	ns
R cerebellum	6.1	-50.7	-12.3	CBL	L cerebellum	-26.3	-69.5	-30.6	CBL	ns	*
L visual associative cortex	-25.9	-63.1	-12.3	VI	L cerebellum	-26.3	-69.5	-30.6	CBL	ns	ns
L secondary visual cortex	-14.7	-84	-13.1	VII	L cerebellum	-26.3	-69.5	-30.6	CBL	ns	ns
L cerebellum	-42.6	-63.7	-46.3	FP	L cerebellum	-21.3	-53.4	-23.6	CBL	ns	ns
L cerebellum	-26.3	-69.5	-30.6	CBL	L cerebellum	-21.3	-53.4	-23.6	CBL	ns	**
L inferior frontal gyrus, orbital part	-46	28.2	-7.1	MF	L caudatum	-14.6	-3.5	21.1	SC	ns	*
L Broca-pars opercularis	-53.1	18.4	10.6	MF	L caudatum	-14.6	-3.5	21.1	SC	ns	*
L pre-supplementary motor area	-46.2	7.9	28.6	FP	L caudatum	-14.6	-3.5	21.1	SC	ns	**

Brain region1	MNI coordinate (x, y, z)			Brain network	Brain region2	MNI coordinate (x, y, z)			Brain network	Follow-up excluded vs baseline MDD	Follow-up excluded vs HCs
L dorsal anterior cingulate cortex	-6	34.1	26.3	MF	L caudatum	-14.6	-3.5	21.1	SC	ns	ns
L angular gyrus	-42.1	-65.6	41.7	FP	L putamen	-24.8	5.6	-0.1	SC	ns	ns
R superior temporal gyrus	61.9	-23.8	-2.8	Mot	L thalamus	-4.9	-10.4	5.8	SC	ns	*
L middle temporal gyrus	-59	-30	3.5	Mot	L thalamus	-4.9	-10.4	5.8	SC	ns	*

Abbreviations and conventions as in Table S1.

Table S3. The overall strength of the sub-network in patients with MDD before and after 6 months of treatment.

	MDD reduced sub-network		MDD increased sub-network	
	mean	SEM	mean	SEM
Baseline MDD	-4.53	0.975	7.136	0.734
Follow-up MDD	2.422	0.888	2.469	0.757
HCs	11.7	2.011	-3.04	1.514

Table S4. In “MDD reduced sub-network”, 42 increased functional connections after 6-month follow-up

Brain region1	MNI coordinate (x, y, z)			Brain network	Brain region2	MNI coordinate (x, y, z)			Brain network
R orbitfrontal gyrus	5.1	34.9	-17.4	DMN	R supramarginal gyrus	59	-29.3	19.5	Mot
R frontal eye field	40.7	14.5	48.2	FP	R angular gyrus	54.2	-45.2	36.9	FP
R Broca-pars opercularis	40	17.6	29.2	FP	R angular gyrus	54.2	-45.2	36.9	FP
R prefrontal lobe	44.6	46.2	-4.9	FP	R fusiform gyrus	60.8	-43.3	-17.6	FP
R primary auditory cortex	59.2	-3.4	2.7	Mot	R primary visual cortex	14.6	-68.3	8.3	VI
R fusiform gyrus	25.2	-44.6	-12.2	VI	R dorsal posterior cingulate cortex	7.8	-23.1	44.9	Mot
R Broca-pars opercularis	55.4	9.6	22.2	FP	R cerebellum	6.1	-50.7	-12.3	CBL
R dorsal posterior cingulate cortex	7.8	-23.1	44.9	Mot	R cerebellum	6.1	-50.7	-12.3	CBL
R pre-supplementary motor area	25.2	12.4	49.4	FP	R cerebellum	7.6	-56.7	-50.8	DMN
R fusiform gyrus	60.8	-43.3	-17.6	FP	L frontal eye field	-5	17.7	48.1	MF
R middle temporal gyrus	50	-33.8	-0.7	MF	L inferior frontal gyrus, orbital part	-46	28.2	-7.1	MF
R superior temporal gyrus	59.3	-43.8	8.5	MF	L inferior frontal gyrus, orbital part	-46	28.2	-7.1	MF
R cerebellum	6.1	-50.7	-12.3	CBL	L Broca-pars opercularis	-53.1	18.4	10.6	MF
R cerebellum	7.2	-69	-20.9	CBL	L Broca-pars opercularis	-53.1	18.4	10.6	MF
L orbitfrontal gyrus	-5.4	29.1	-10.1	DMN	L insula	-37.8	-12.9	-1.4	Mot
R secondary visual cortex	7.8	-88.6	1.9	VI	L primary sensory area	-50.6	-23.8	41.4	Mot
R cerebellum	7.6	-56.7	-50.8	DMN	L visual motor area	-9.8	-66.3	55.1	SAL
L dorsolateral prefrontal lobe	-27.3	34.1	36.4	SAL	L visual motor area	-9.8	-66.3	55.1	SAL
R visual associative cortex	21	-63.7	-9	VI	L middle temporal gyrus	-59	-30	3.5	Mot
R dorsolateral prefrontal lobe	37.6	35.4	31.1	SAL	L fusiform gyrus	-60.4	-50	-14	FP
L visual motor area	-9.8	-66.3	55.1	SAL	L visual associative cortex	-41.3	-75.4	22.8	DMN
R dorsal posterior cingulate cortex	7.8	-23.1	44.9	Mot	L visual associative cortex	-17	-50.7	0.8	VI
L middle temporal gyrus	-59	-30	3.5	Mot	L secondary visual cortex	-8.9	-70.7	-1.7	VI

Brain region1	MNI coordinate (x, y, z)			Brain network	Brain region2	MNI coordinate (x, y, z)			Brain network
R visual motor area	14.8	-68.4	34.9	VI	L ventral posterior cingulate cortex	-5	-36	32	DMN
R visual motor area	14.8	-68.4	34.9	VI	L ventral posterior cingulate cortex	-7.4	-18.2	30	SAL
pre-supplementary motor area	7	-8.1	52.9	Mot	L amygdala	-26.8	2.4	-18.7	Mot
L dorsolateral prefrontal lobe	-27.3	34.1	36.4	SAL	L hippocampus	-21.5	-36.9	5.8	SC
L visual motor area	-9.8	-66.3	55.1	SAL	L hippocampus	-21.5	-36.9	5.8	SC
L ventral posterior cingulate cortex	-6.5	-53.9	37.4	DMN	L hippocampus	-35.7	-24.8	-14.9	SC
R pre-supplementary motor area	25.2	12.4	49.4	FP	L cerebellum	-8.7	-55.2	-52.1	DMN
L visual motor area	-9.8	-66.3	55.1	SAL	L cerebellum	-8.7	-55.2	-52.1	DMN
R angular gyrus	41.4	-75.3	28	DMN	L cerebellum	-40.3	-74.2	-29.2	VII
L angular gyrus	-42.1	-65.6	41.7	FP	L cerebellum	-40.3	-74.2	-29.2	VII
R dorsolateral prefrontal lobe	48.3	35.7	15.2	FP	L cerebellum	-6.5	-50.1	-11.4	SAL
R frontal eye field	40.7	14.5	48.2	FP	L cerebellum	-42.6	-63.7	-46.3	FP
L Broca-pars opercularis	-53.1	18.4	10.6	MF	L cerebellum	-8	-68.4	-19.9	CBL
L pre-supplementary motor area	-23.2	10.7	53.6	FP	L cerebellum	-24.3	-37.8	-44.3	CBL
R visual associative cortex	21	-63.7	-9	VI	L putamen	-24.8	5.6	-0.1	SC
R pre-supplementary motor area	7	-8.1	52.9	Mot	L thalamus	-11.6	-25.6	14.8	SC
R ventral anterior cingulate cortex	5.3	-1	35.6	Mot	L thalamus	-11.6	-25.6	14.8	SC
L pre-supplementary motor area	-27.6	-9.1	55.9	Mot	L thalamus	-11.6	-25.6	14.8	SC
L dorsal posterior cingulate cortex	-9	-42.6	50.1	SAL	L thalamus	-11.6	-25.6	14.8	SC

Abbreviations and conventions as in Table S1.

Table S5. In "MDD increased sub-network", 31 decreased functional connections after 6-month follow-up

Brain region1	MNI coordinate (x, y, z)			Brain network	Brain region2	MNI coordinate (x, y, z)			Brain network
R orbitfrontal gyrus	5.1	34.9	-17.4	DMN	R frontal eye field	40.7	14.5	48.2	FP
R prefrontal lobe	8.2	45.9	-1.7	DMN	R frontal eye field	40.7	14.5	48.2	FP
R pre-supplementary motor area	6	-22.3	65.6	Mot	R primary sensory area	32.4	-39.2	49.6	Mot
R primary sensory area	32.4	-39.2	49.6	Mot	R visual motor area	7.5	-57.3	61.8	SAL
R pre-supplementary motor area	13.7	6.3	65.4	SAL	R visual associative cortex	21	-63.7	-9	VI
R visual motor area	7.5	-57.3	61.8	SAL	R dorsal posterior cingulate cortex	7.8	-23.1	44.9	Mot
R frontal eye field	40.7	14.5	48.2	FP	L prefrontal lobe	-6.9	48.3	-5.7	DMN
R dorsolateral prefrontal lobe	37.6	35.4	31.1	SAL	L prefrontal lobe	-6	48.1	11.7	MF
R dorsolateral prefrontal lobe	48.3	35.7	15.2	FP	L prefrontal lobe	-6	48.1	11.7	MF
R dorsal posterior cingulate cortex	7.8	-23.1	44.9	Mot	L prefrontal lobe	-28.8	50.1	21.7	SAL
L orbitfrontal gyrus	-5.4	29.1	-10.1	DMN	L prefrontal lobe	-28.8	50.1	21.7	SAL
L inferior frontal gyrus, orbital part	-46	28.2	-7.1	MF	L pre-supplementary motor area	-23.2	10.7	53.6	FP
L orbitfrontal gyrus	-5.4	29.1	-10.1	DMN	L pre-supplementary motor area	-27.6	-9.1	55.9	Mot
R primary sensory area	32.4	-39.2	49.6	Mot	L visual motor area	-9.8	-66.3	55.1	SAL
L pre-supplementary motor area	-58.1	-5.6	27.2	Mot	L visual motor area	-9.8	-66.3	55.1	SAL
L visual motor area	-25.4	-54.8	64.1	Vas	L visual motor area	-9.8	-66.3	55.1	SAL
R cerebellum	39.1	-74.9	-29.7	VII	L supramarginal gyrus	-59.5	-25.9	21.9	Mot
R orbitfrontal gyrus	5.1	34.9	-17.4	DMN	L angular gyrus	-42.1	-65.6	41.7	FP
L orbitfrontal gyrus	-18.2	19.1	-21	SAL	L angular gyrus	-42.1	-65.6	41.7	FP
L inferior frontal gyrus, orbital part	-28.4	36	-15.6	SAL	L angular gyrus	-42.1	-65.6	41.7	FP
R orbitfrontal gyrus	5.1	34.9	-17.4	DMN	L visual associative cortex	-41.3	-75.4	22.8	DMN
L orbitfrontal gyrus	-5.4	29.1	-10.1	DMN	L visual associative cortex	-41.3	-75.4	22.8	DMN
L visual motor area	-9.8	-66.3	55.1	SAL	L pre-supplementary motor area	-7.8	-22.4	46.1	Mot

Brain region1	MNI coordinate (x, y, z)			Brain network	Brain region2	MNI coordinate (x, y, z)			Brain network
L dorsolateral prefrontal lobe	-10.2	55.7	30.2	MF	L dorsal anterior cingulate cortex	-6	34.1	26.3	MF
L frontal eye field	-11.2	34.3	51.5	MF	L dorsal anterior cingulate cortex	-6	34.1	26.3	MF
R supramarginal gyrus	59	-29.3	19.5	Mot	L cerebellum	-40.3	-74.2	-29.2	VII
R cerebellum	6.1	-50.7	-12.3	CBL	L cerebellum	-42.6	-63.7	-46.3	FP
L cerebellum	-6.5	-50.1	-11.4	SAL	L cerebellum	-42.6	-63.7	-46.3	FP
R secondary visual cortex	17.9	-83.4	-11.3	VII	L cerebellum	-10.3	-81.2	-32.3	FP
R secondary visual cortex	17.9	-83.4	-11.3	VII	L cerebellum	-8	-68.4	-19.9	CBL
R secondary visual cortex	17.9	-83.4	-11.3	VII	L cerebellum	-26.3	-69.5	-30.6	CBL

Abbreviations and conventions as in Table S1.

Table S6. In "follow-up increased sub-network", 64 increased functional connections

Brain region1	MNI coordinate (x, y, z)			Brain network	Brain region2	MNI coordinate (x, y, z)			Brain network
R orbitfrontal gyrus	5.1	34.9	-17.4	DMN	R pre-supplementary motor area	6	-22.3	65.6	Mot
R orbitfrontal gyrus	5.1	34.9	-17.4	DMN	R primary sensory area	20	-33.2	69.8	Mot
R prefrontal lobe	8.2	45.9	-1.7	DMN	R primary sensory area	43.3	-10.8	13.9	Mot
R orbitfrontal gyrus	5.1	34.9	-17.4	DMN	R supramarginal gyrus	59	-29.3	19.5	Mot
R prefrontal lobe	8.2	45.9	-1.7	DMN	R supramarginal gyrus	59	-29.3	19.5	Mot
R pre-supplementary motor area	6.1	14	48.7	SAL	R angular gyrus	41.4	-75.3	28	DMN
R orbitfrontal gyrus	5.1	34.9	-17.4	DMN	R primary auditory cortex	59.2	-3.4	2.7	Mot
R prefrontal lobe	8.2	45.9	-1.7	DMN	R primary auditory cortex	59.2	-3.4	2.7	Mot
R orbitfrontal gyrus	5.1	34.9	-17.4	DMN	R dorsal posterior cingulate cortex	7.8	-23.1	44.9	Mot
R dorsolateral prefrontal lobe	48.3	35.7	15.2	FP	R secondary visual cortex	14.6	-46	2.8	VI
R visual associative cortex	18.9	-81.8	41.5	VI	R cerebellum	32.2	-78.5	-40.4	VII
R dorsolateral prefrontal lobe	48.3	35.7	15.2	FP	R cerebellum	6.1	-50.7	-12.3	CBL
R pre-supplementary motor area	6.1	14	48.7	SAL	R cerebellum	6.1	-50.7	-12.3	CBL
R pre-supplementary motor area	39.7	3.4	34	FP	R cerebellum	11.7	84.1	-34.7	FP
R angular gyrus	41.4	-75.3	28	DMN	R cerebellum	11.7	84.1	-34.7	FP
R visual associative cortex	18.9	-81.8	41.5	VI	R cerebellum	11.7	84.1	-34.7	FP
R angular gyrus	41.4	-75.3	28	DMN	R cerebellum	41.9	-64	-49.2	FP
R ventral posterior cingulate cortex	7	-18.8	29.8	SAL	R cerebellum	7.1	-53.7	-34.4	CBL
R insula	41.8	5	-7.6	Mot	R caudatum	12.6	20.2	-0.7	SC
R secondary visual cortex	7.8	-88.6	1.9	VI	R caudatum	12.6	20.2	-0.7	SC
R brainstem	19.3	-7.7	-14.8	SC	R caudatum	12.6	20.2	-0.7	SC
R cerebellum	30.4	-36.4	-31.1	SAL	R caudatum	12.6	20.2	-0.7	SC
R insula	41.8	5	-7.6	Mot	L orbitfrontal gyrus	-8.2	39.7	-21.4	MF

Brain region1	MNI coordinate (x, y, z)			Brain network	Brain region2	MNI coordinate (x, y, z)			Brain network
R cerebellum	6.1	-50.7	-12.3	CBL	L prefrontal lobe	-29.9	54.3	0.5	FP
R cerebellum	36.7	-57.1	-32.8	CBL	L prefrontal lobe	-29.9	54.3	0.5	FP
R visual motor area	14.8	-68.4	34.9	VI	L dorsolateral prefrontal lobe	-27.3	34.1	36.4	SAL
R supramarginal gyrus	59	-29.3	19.5	Mot	L dorsolateral prefrontal lobe	-27.3	34.1	36.4	SAL
R cerebellum	6.1	-50.7	-12.3	CBL	L pre-supplementary motor area	-46.2	7.9	28.6	FP
R cerebellum	7.1	-53.7	-34.4	CBL	L pre-supplementary motor area	-46.2	7.9	28.6	FP
R orbitfrontal gyrus	5.1	34.9	-17.4	DMN	L pre-supplementary motor area	-57	-3.4	6.8	Mot
R prefrontal lobe	8.2	45.9	-1.7	DMN	L pre-supplementary motor area	-57	-3.4	6.8	Mot
R caudatum	12.6	20.2	-0.7	SC	L pre-supplementary motor area	-57	-3.4	6.8	Mot
R caudatum	12.6	20.2	-0.7	SC	L insula	38.7	8.1	-4.8	SAL
R caudatum	12.6	20.2	-0.7	SC	L insula	-37.8	-12.9	-1.4	Mot
R secondary visual cortex	7.8	-88.6	1.9	VI	L primary sensory area	-50.6	-23.8	41.4	Mot
R caudatum	12.6	20.2	-0.7	SC	L primary sensory area	-41.2	-15.6	14.5	Mot
L orbitfrontal gyrus	-5.4	29.1	-10.1	DMN	L primary sensory area	-41.2	-15.6	14.5	Mot
L orbitfrontal gyrus	-8.2	39.7	-21.4	MF	L supramarginal gyrus	-42.2	-31.2	15.9	Mot
R orbitfrontal gyrus	5.1	34.9	-17.4	DMN	L supramarginal gyrus	-59.5	-25.9	21.9	Mot
R prefrontal lobe	8.2	45.9	-1.7	DMN	L supramarginal gyrus	-59.5	-25.9	21.9	Mot
R dorsolateral prefrontal lobe	8.4	53.3	23.9	MF	L supramarginal gyrus	-59.5	-25.9	21.9	Mot
R caudatum	12.6	20.2	-0.7	SC	L supramarginal gyrus	-59.5	-25.9	21.9	Mot
R ventral posterior cingulate cortex	7	-18.8	29.8	SAL	L inferior temporal gyrus	-37.8	-13.2	-29.3	Mot
R cerebellum	11.7	84.1	-34.7	FP	L visual associative cortex	-31.6	-87.2	12.5	VAs
R supramarginal gyrus	59	-29.3	19.5	Mot	L visual associative cortex	-17	-50.7	0.8	VI
R dorsal posterior cingulate cortex	8.3	-39.9	48.1	SAL	L visual associative cortex	-17	-50.7	0.8	VI
L supramarginal gyrus	-59.5	-25.9	21.9	Mot	L secondary visual cortex	-8.9	-70.7	-1.7	VI

Brain region1	MNI coordinate (x, y, z)			Brain network	Brain region2	MNI coordinate (x, y, z)			Brain network
L inferior temporal gyrus	-49.3	-4.7	-37.4	MF	L pre-supplementary motor area	-7.8	-22.4	46.1	Mot
R caudatum	12.6	20.2	-0.7	SC	L ventral anterior cingulate cortex	-5.1	13.2	28.7	SAL
R dorsolateral prefrontal lobe	48.3	35.7	15.2	FP	L hippocampus	-32.1	-40.2	-4	SC
R Broca-pars opercularis	55.4	9.6	22.2	FP	L hippocampus	-32.1	-40.2	-4	SC
R dorsolateral prefrontal lobe	48.3	35.7	15.2	FP	L cerebellum	-6.5	-50.1	-11.4	SAL
R Broca-pars opercularis	55.4	9.6	22.2	FP	L cerebellum	-6.5	-50.1	-11.4	SAL
R pre-supplementary motor area	39.7	3.4	34	FP	L cerebellum	-6.5	-50.1	-11.4	SAL
L pre-supplementary motor area	-46.2	7.9	28.6	FP	L cerebellum	-6.5	-50.1	-11.4	SAL
R dorsolateral prefrontal lobe	48.3	35.7	15.2	FP	L cerebellum	-27.8	-36	-30.9	SAL
L inferior frontal gyrus, orbital part	-28.4	36	-15.6	SAL	L cerebellum	-27.8	-36	-30.9	SAL
R angular gyrus	41.4	-75.3	28	DMN	L cerebellum	-10.3	-81.2	-32.3	FP
R dorsolateral prefrontal lobe	48.3	35.7	15.2	FP	L cerebellum	-21.3	-53.4	-23.6	CBL
R caudatum	12.6	20.2	-0.7	SC	L cerebellum	-21.3	-53.4	-23.6	CBL
R insula	41.8	5	-7.6	Mot	L caudatum	-10.4	10.9	-8.1	SC
R secondary visual cortex	7.8	-88.6	1.9	VI	L caudatum	-10.4	10.9	-8.1	SC
R dorsal posterior cingulate cortex	8.3	-39.9	48.1	SAL	L caudatum	-10.4	10.9	-8.1	SC
L pre-supplementary motor area	-7.8	-22.4	46.1	Mot	L caudatum	-10.4	10.9	-8.1	SC

Abbreviations and conventions as in Table S1.

Table S7. In “follow-up decreased sub-network”, 36 decreased functional connections

Brain region1	MNI coordinate (x, y, z)			Brain network	Brain region2	MNI coordinate (x, y, z)			Brain network
R inferior frontal gyrus, orbital part	53.6	24.8	0.9	MF	R Broca-pars triangularis	37	20.8	5.9	SAL
R inferior frontal gyrus, orbital part	53.6	24.8	0.9	MF	R insula	41.4	3.5	7.2	Mot
R precuneus	7.5	-57.3	61.8	SAL	R supramarginal gyrus	52.8	-27.3	40.9	Mot
R Broca-pars opercularis	55.4	9.6	22.2	FP	R supramarginal gyrus	59	-29.3	19.5	Mot
R insula	41.4	3.5	7.2	Mot	R angular gyrus	54.2	-45.2	36.9	FP
R supramarginal gyrus	59	-29.3	19.5	Mot	R angular gyrus	54.2	-45.2	36.9	FP
R angular gyrus	41.4	-75.3	28	DMN	R temporal pole	52.8	10.9	-21.8	MF
R Broca-pars opercularis	55.4	9.6	22.2	FP	R primary auditory cortex	59.2	-3.4	2.7	Mot
R angular gyrus	54.2	-45.2	36.9	FP	R primary auditory cortex	59.2	-3.4	2.7	Mot
R angular gyrus	41.4	-75.3	28	DMN	R secondary visual cortex	7.7	-75	25	VI
R inferior frontal gyrus, orbital part	53.6	24.8	0.9	MF	R ventral anterior cingulate cortex	5.3	-1	35.6	Mot
R precuneus	7.5	-57.3	61.8	SAL	R dorsal posterior cingulate cortex	7.8	-23.1	44.9	Mot
R dorsal posterior cingulate cortex	7.8	-23.1	44.9	Mot	R dorsal posterior cingulate cortex	8.3	-39.9	48.1	SAL
R dorsal posterior cingulate cortex	7.8	-23.1	44.9	Mot	L visual motor area	-7.4	-34.1	67.5	Mot
R supramarginal gyrus	52.8	-27.3	40.9	Mot	L precuneus	-9.8	-66.3	55.1	SAL
R angular gyrus	54.2	-45.2	36.9	FP	L supramarginal gyrus	-59.5	-25.9	21.9	Mot
R fusiform gyrus	55.2	-56.3	-4.8	VAs	L supramarginal gyrus	-59.5	-25.9	21.9	Mot
R fusiform gyrus	60.8	-43.3	-17.6	FP	L supramarginal gyrus	-59.5	-25.9	21.9	Mot
R primary auditory cortex	59.2	-3.4	2.7	Mot	L angular gyrus	-53.4	-43.5	38.8	FP
L supramarginal gyrus	-59.5	-25.9	21.9	Mot	L angular gyrus	-53.4	-43.5	38.8	FP
R orbitfrontal gyrus	5.1	34.9	-17.4	DMN	L visual associative cortex	-41.3	-75.4	22.8	DMN
R temporal pole	52.8	10.9	-21.8	MF	L visual associative cortex	-41.3	-75.4	22.8	DMN
R primary auditory cortex	39.9	-25.6	14.4	Mot	L visual associative cortex	-41.3	-75.4	22.8	DMN

Brain region1	MNI coordinate (x, y, z)			Brain network	Brain region2	MNI coordinate (x, y, z)			Brain network
R temporal pole	52.8	10.9	-21.8	MF	L visual associative cortex	-16.8	-84.9	33	VI
R angular gyrus	41.4	-75.3	28	DMN	L primary visual cortex	-6	-81.2	12.2	VI
R pre-supplementary motor area	32.1	-5.4	52.1	SAL	L pre-supplementary motor area	-7.8	-22.4	46.1	Mot
R precuneus	7.5	-57.3	61.8	SAL	L pre-supplementary motor area	-7.8	-22.4	46.1	Mot
L angular gyrus	-53.4	-43.5	38.8	FP	L pre-supplementary motor area	-7.8	-22.4	46.1	Mot
R Broca-pars opercularis	55.4	9.6	22.2	FP	L ventral anterior cingulate cortex	3.8	-5.1	32.6	SAL
R angular gyrus	54.2	-45.2	36.9	FP	L ventral anterior cingulate cortex	3.8	-5.1	32.6	SAL
R dorsal posterior cingulate cortex	7.8	-23.1	44.9	Mot	L dorsal posterior cingulate cortex	-9	-42.6	50.1	SAL
R angular gyrus	41.4	-75.3	28	DMN	L amygdala	-26.8	2.4	-18.7	Mot
R orbitfrontal gyrus	5.1	34.9	-17.4	DMN	L cerebellum	-30.2	-80.2	-40.4	FP
R orbitfrontal gyrus	5.1	34.9	-17.4	DMN	L cerebellum	-10.3	-81.2	-32.3	FP
L visual motor area	-7.4	-34.1	67.5	Mot	L cerebellum	-10.3	-81.2	-32.3	FP
R angular gyrus	41.4	-75.3	28	DMN	L caudatum	-10.4	10.9	-8.1	SC

Abbreviations and conventions as in Table S1.
